# Biomechanics of haemostasis and thrombosis in health and disease: from the macro- to molecular scale

**DOI:** 10.1111/jcmm.12041

**Published:** 2013-03-14

**Authors:** Reginald Tran, David R Myers, Jordan Ciciliano, Elaissa L Trybus Hardy, Yumiko Sakurai, Byungwook Ahn, Yongzhi Qiu, Robert G Mannino, Meredith E Fay, Wilbur A Lam

**Affiliations:** a Department of Pediatrics, Division of Pediatric Hematology/Oncology, Aflac Cancer Center and Blood Disorders Service of Children's Healthcare of Atlanta, Emory University School of MedicineAtlanta, Georgia, USA; b Wallace H. Coulter Department of Biomedical Engineering, Georgia Institute of Technology and Emory UniversityAtlanta, Georgia, USA; c Parker H. Petit Institute of Bioengineering & Bioscience, Georgia Institute of TechnologyAtlanta, Georgia, USA; d Winship Cancer Institute of Emory UniversityAtlanta, Georgia, USA

**Keywords:** cellular mechanics, biomechanics, haemostasis, thrombosis, mechanobiology, mechanotransduction

## Abstract

Although the processes of haemostasis and thrombosis have been studied extensively in the past several decades, much of the effort has been spent characterizing the biological and biochemical aspects of clotting. More recently, researchers have discovered that the function and physiology of blood cells and plasma proteins relevant in haematologic processes are mechanically, as well as biologically, regulated. This is not entirely surprising considering the extremely dynamic fluidic environment that these blood components exist in. Other cells in the body such as fibroblasts and endothelial cells have been found to biologically respond to their physical and mechanical environments, affecting aspects of cellular physiology as diverse as cytoskeletal architecture to gene expression to alterations of vital signalling pathways. In the circulation, blood cells and plasma proteins are constantly exposed to forces while they, in turn, also exert forces to regulate clot formation. These mechanical factors lead to biochemical and biomechanical changes on the macro- to molecular scale. Likewise, biochemical and biomechanical alterations in the microenvironment can ultimately impact the mechanical regulation of clot formation. The ways in which these factors all balance each other can be the difference between haemostasis and thrombosis. Here, we review how the biomechanics of blood cells intimately interact with the cellular and molecular biology to regulate haemostasis and thrombosis in the context of health and disease from the macro- to molecular scale. We will also show how these biomechanical forces in the context of haemostasis and thrombosis have been replicated or measured *in vitro*.

- Introduction
- Macroscale Biomechanics in Haemostasis
  – Tools for Macroscale Study
  – Haemostatic diseases & correlations withmacroscale mechanics
  – Macroscale mechanical clot changes associated with bleeding disorders
- Microscale Biomechanics in Haemostasis
  – Tools for Microscale Measurements
  – Mechanotransduction of Platelets in Haemostasis
  – Haemostatic Diseases & Correlations Related to Microscale Phenomena
  – Microscale changes are also observed for various bleeding disorders
  – Biochemical and Biomechanical Interactions
- Molecular-Scale Biomechanics in Haemostasis
  – Molecular Components Involved in Haemostasis
  – Molecular-scale changes in mechanics associated with bleeding disorders
- Erythrocytes in Haemostasis
  – Erythrocyte stiffness is varied and affected by a number of different diseases
- Concluding Statements

## Introduction

Mechanical forces have been observed to affect the biological functions of cells for centuries. However, owing to technological limitations and limited knowledge of cell physiology, only qualitative observations were able to be made. The last few decades has greatly accelerated the development of the field of cell biomechanics with the creation of tools that allowed researchers to measure and induce forces on both bulk tissues/cell suspensions and individual cells. From these studies, the field has discovered that mechanics has a profound impact on important cellular functions such as cell adhesion, migration, differentiation and proliferation. Alternations in the mechanical properties of cells have also been associated with clinical manifestations and implications. Many differences in mechanical properties or mechanical regulations have been identified between healthy and disease states. Chondrocytes are found to be stiffer in arthritis, epithelial cells and fibroblasts have been shown to be softer in metastasis and airway smooth muscle cells show increased stiffening and reduced contractility in asthma patients 1. The most clinically significant example of how mechanics affects disease involves atherosclerosis and cardiovascular disease. In particular, the haemodynamic forces imposed on endothelial cells from blood flow stimulate the release of vasodilatory and vasoconstrictive biochemical factors, change gene expression, impact cell metabolism and regulate the overall cell morphology 2, 3. When endothelial cells are exposed to irregular flow patterns that cause the endothelium to be exposed to low and oscillatory shear stress, strong correlations have been made with endothelial cell dysfunction. These events in turn lead to increased lipoprotein uptake, increased leucocyte adhesion and monocyte, macrophage and smooth muscle cell proliferation, which may contribute to atherosclerosis.

Blood cells are also exposed to the same mechanical haemodynamic environment as endothelial cells. Therefore, blood cell physiology is also likely regulated by mechanical forces at some level, which has implications for the mechanical regulation of haemostasis, the process of clotting to prevent blood loss following vascular injury. Biomechanical changes in the blood cells themselves can also ultimately affect the rheological properties of blood, which subsequently lead to force modulation. The various cellular and soluble factors involved with clot formation have been identified and well characterized biologically and biochemically, but the whole picture is still incomplete. Our current knowledge of haemostasis consists of a two-phase process that involves (i) platelet aggregation mediated by surface receptors and soluble ligands and (ii) a series of biochemical factors that comprise the coagulation cascade to ultimately form a mesh-like fibrin network. Platelets possess membrane receptors similar to other types of cells to sense the environment. During vascular injury, platelets are activated by binding to the exposed adhesive proteins such as collagen and von Willebrand Factor (vWF), and by binding of soluble agonists, such as ADP, thrombin and thromboxane A2. These processes trigger signalling cascades, causing cytoskeleton reorganization, shape changes and granule secretion, and these signalling pathways converge to the ‘inside-out’ activation of surface receptor GP IIb/IIIa, also known as the platelet αIIbβ3 integrin. Binding of activated GP IIb/IIIa to adhesive proteins such as fibrinogen, fibronectin, and vWF, followed by clustering of receptors on the membrane, induces an ‘outside-in’ signalling pathway to further amplify the activation of platelets, leading to platelet aggregation, contraction and clot formation. Occurring simultaneously with platelet aggregation is the coagulation pathway, which involves the activation of various enzymes, which in turn, subsequently activate other specific enzymes, thus amplifying the coagulation reaction. This ultimately leads to the cross-linking of fibrinogen into fibrin polymers to form a fibrin mesh. Ultimately, the role of haemostasis is mechanical in nature, as the purpose is to prevent blood loss from the high pressure environment, the circulation, to the lower pressure outside environment. Shear stress, which is caused by fluid friction in flowing blood, has also been shown to regulate the release of antithrombotic factors from endothelial cells, which demonstrates that coagulation is at least indirectly regulated by mechanical forces 4. This review summarizes the existing published work that characterizes the interactions between mechanics and clot formation at different size scales.

**Fig. 1:**
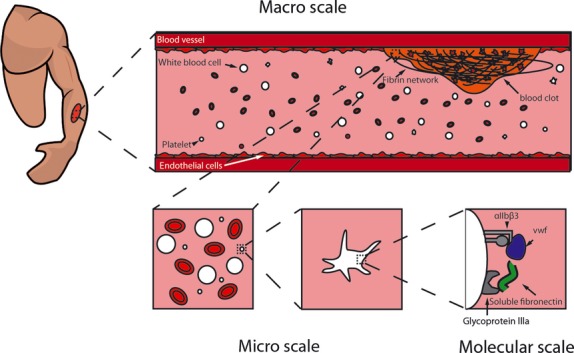
*In vivo* cartoon representation of the multiple scales at which haemostasis occurs.

Together, platelets and soluble clotting factors work in concert at multiple scales to allow for platelet aggregation and formation of the fibrin network to create a stable clot that can eventually be degraded once healing has occurred. This review highlights work that has investigated the role of biomechanics in haemostasis and thrombosis in health and disease at the macro-, micro- and molecular scale. Here, the macroscale is defined as any interactions that occur over a scale greater than 1 mm. This section will incorporate aspects of bulk cells, interactions with whole clots, and blood rheology. The microscale is defined as any interaction that occurs or elicits a change which takes place on a scale less than 1 mm, but greater than 1 μm. This section will typically cover biomechanics at the cellular level. Finally, the molecular scale is defined as any interaction that occurs on a scale less than 1 μm. This section will cover the biomechanics of individual molecular components of blood involved in haemostasis such as vWF and fibrin polymers. The scale of the forces that have a role in haemostasis and thrombosis can be visualized by the schematic shown in Figure 1. The tools and equipment used to either measure or induce these forces experimentally are organized in Table 1, which includes the type of force, the scale and the findings from experiments that used the tool. The role of erythrocytes in haemostasis will be discussed in a separate section as recent research suggests that erythrocytes may indirectly contribute to haemostasis by concentrating platelets towards the vessel wall for a faster response during vascular injury 5.

**Table 1:** General overview of techniques used to study haemostasis

Technique	Scale	Type of force	Findings	References
Rheometry	Macroscale	Input: Shear	Shear stress positively regulates the elastic modulus of clots, which is a material property that characterizes how much an object deforms under applied force without permanently deforming (*i.e*. material stiffness)	18, 30–32
		Output: Clot elasticity	Cellular components affect the elastic modulus of clots	
			Platelets have a positive relationship with the elastic modulus whereas high RBC concentrations have a biphasic effect on clot elasticity, increasing it at low concentrations and decreasing at higher concentrations	
			The elastic modulus of clots is also positively regulated by fibrinogen concentration and GPIIb/IIIa receptor activity	
Thromboelastography	Macroscale	Input: Shear	Clot strength (MA values denoting how much force can be applied before a clot ruptures) increased with platelet activation and platelet concentration. Inhibition of the GPIIb/IIIa receptor significantly decreases clot strength	19–25
		Output: Clot strength, clotting time	In disease, critically ill patients with sepsis, women with mild pre-eclampsia, women with recurrent miscarriage, colorectal and breast cancer had higher clot strength compared with controls	
			Women with severe pre-eclampsia with thrombocytopenia had significantly lower clot strength compared with controls. Patients who had higher MA values pre-surgery were more likely to have thrombotic complications after surgery.	
Microfluidics	Microscale	Input: Shear + hydrodynamic force	Microgradients formed by stenoses cause discoid platelets to preferentially adhere to low-shear zones at the downstream face of forming thrombi	86, 117, 122
		Output: Platelet aggregation or biochemical response	The stabilization of platelet aggregates is also dependent on the dynamic restructuring of membrane tethers	
			RBCs under shear release ATP with a time delay	
Atomic Force Microscopy	Microscale	Input: Precise compressive or tensile force	Platelets have an average maximum contractile force of 29 nN, and can exert forces up to 70 nN	84, 85, 102, 119
		Output: Deformation of cell, contraction or adhesive force	The stiffness of the platelet binding substrate may also positively modulate platelet contraction force	
			The elastic modulus of RBCs varies between normal, oxygenated sickle and deoxygenated sickle cells with deoxygenated sickle cells being the stiffest	
			Oligomer unfolding gives rise to a periodic sawtooth pattern with an average peak force of 94 pN that can be attributed to the unfolding of the triple helix coiled coils	
			Extensibility of fibrin starts with the α-helical coiled coils which serve as molecular capacitors that extend and contract reversibly, followed by force-induced β strand release, and the eventual dissociation of the γ nodule	
Optical Tweezers	Microscale	Input: Precise compressive or tensile force	Tensile forces of 7–14 pN can unfold a single A2 domain of vWF. These forces contribute to the mechanical properties and stability of vWF	99, 100
	Molecular scale	Output: Deformation of cell, contraction or adhesive force		

## Macroscale biomechanics in haemostasis

At its core, the main purpose of haemostasis is very mechanical in nature, and is analogous to patching a tire that has been punctured. Independent of the biochemical aspects of clot formation, the blood clot must ultimately be mechanically stable enough to mitigate blood loss. To achieve this, there are several mechanical forces that act on bulk blood to regulate the process of haemostasis at the macroscale. These blood cells exist in an extremely dynamic environment as they constantly circulate through the vasculature for the entirety of their lifespans. The major mechanical stimuli acting on these cells include shear stress caused by fluid friction and hydrodynamic forces exerted on the cells by the moving fluid. However, to exist in such an environment, the clots formed by these cells must themselves be mechanically stable to form a functional plug while avoiding increased stiffness such that clot dissolution cannot occur once the wound is healed.

### Tools for macroscale study

**Fig. 2:**
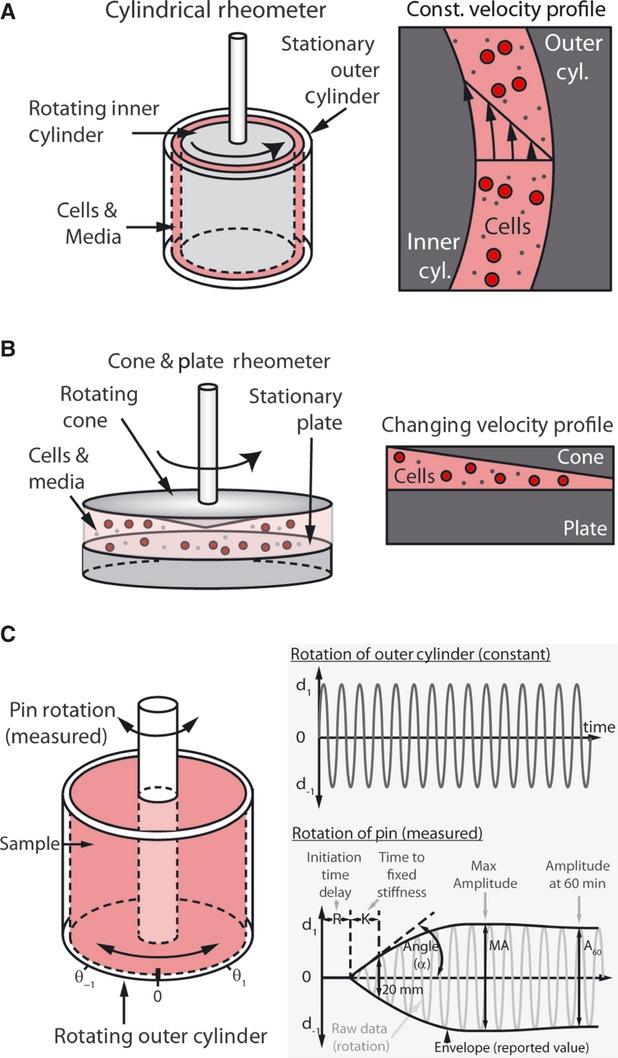
(**A**) A cylindrical rheometer consists of a stationary outer cylinder and a rotating inner cylinder to shear cells in suspension. This allows for controlled, continuous Couette flow, which has a constant velocity profile. Couette flow is fluid movement generated by the movement of a flat plate relative to a stationary plate. The layer of fluid in contact with the cylinders is constrained to the respective surfaces, resulting in the velocity profile shown above where it is zero at the stationary outer cylinder and maximal with the same velocity of the inner moving cylinder. (**B**) Similar to the cylindrical rheometer, a rotating cone stationed about a stationary plate allows for a varying velocity profile as the distance from the tip of the cone increases. (**C**) Thromboelastography works on a similar principle as the rheometer, but instead plots the torque of a rotating pin relative to a rotating outer cylinder to fully characterize clot formation as it occurs. An initial time delay is observed before fibrin begins to form, characterized by the R time. K and α are kinetic parameters that characterize the time to stable clot formation. As the clot becomes stiffer and the pin becomes more coupled with the oscillating cylinder, the trace increases in amplitude until maximal amplitude (MA) is reached, denoting the maximum clot firmness. Once fibrinolysis or clot retraction occurs, the amplitude starts decreasing again as the pin and cylinder start rotating out of phase.

During the last several decades, numerous different types of equipment have been used to recapitulate the mechanical environment *in vitro* to observe macroscale changes. This section will specifically consider rheometry, thromboelastography, ektacytometry and bulk platelet contraction. Rheometry, thromboelastography and ektacytometry allow the application of controlled continuous or oscillatory shear stress. Using oscillatory shear stress enables the measurement of the complex modulus of elasticity. Bulk platelet contraction provides an estimate of the forces applied collectively by a population of platelets. All these parameters have been important in drawing a link between the mechanical response of clot formation to mechanical stimuli and biochemical factors. However, each technique has different advantages and disadvantages which have provided different insights into the biophysical aspects of haemostasis. These techniques are illustrated below and are briefly described below in Figure 2.

Rheometry takes advantage of tools originally developed to measure viscous properties of fluids and uses them to apply shear stress to bulk cells. Blood or a suspension of cells is placed in between a moving and non-moving surface to create Couette flow, which is fluid movement that occurs as a result of liquids bounded between a moving wall and a stationary wall. Typical set-ups include sandwiching the liquid between two cylinders (where the inner cylinder rotates), or between two round, flat plates (in which the top plate rotates). By inducing Couette flow in the liquid, the velocity will linearly increase from zero at the stationary plate to the velocity of the moving plate. The cone and plate rheometer is similar to the other ones mentioned above, but a homogeneous shear stress is applied owing to a linear change in fluid velocity and height 6.

These tools each allow the application of controlled continuous or oscillatory shear stress. Using oscillatory shear stress enables the measurement of the complex modulus of elasticity. One plate is driven in an oscillatory manner, and the other plate is constrained with a torsion bar of known value and measures the response. As the material couples the oscillating plate to the measurement plate, any differences in the motion of the two plates will be related to the material properties of the substance. An infinitely stiff material would couple the plates together such that there is no difference between the driving plate and measuring plate. However, as is the case for most materials, the measurement plate will not turn with the same amplitude and will move with some time delay. These are correlated with the storage modulus of elasticity, G', and the loss modulus (dynamic viscosity), G'', can be calculated.

#### Thromboelastography (TEG)

Thromboelastography utilizes similar principles to rheometry and ektacytometry and specifically monitors the clotting of a sample of blood, first described by Hartet 7. Here, the main ideas of thromboelastography are highlighted, and the interested reader is directed to a number of excellent reviews 8–10. Thromboelastography uses a cylindrical cuvette 11 which is rotated back and forth at a standardized rate. A small pin is inserted into the sample, and the rotation of this pin is monitored. Over time, as the clot forms, the movement of the pin becomes coupled into that of the cuvette. The raw data of the pin movement is a sinusoidal curve with constant period and changing amplitude. A thromboelastograph presents the final maximum amplitudes of the pin over time. Ultimately, this graph provides information on the rate of clot formation and the elasticity of the clot, as well as clot lysis under low shear conditions. Rotation Thromboelastometry (ROTEM) is a similar technique in which the pin is rotated by a spring and the motion is monitored optically. As a constant force is applied by the spring (rather than a constant applied motion), the motion will decrease as a clot forms.

In a standard thromboelastograph, several measurements have been identified and standardized to assist in comparing the graphs (Fig. 2C). The R time is how long it takes the pin to begin rotating up to 2 mm, and represents the clotting time. The K time and α (angle) are related to the clotting kinetics. The K time is how long it takes the pin movement to go from 2 to 20 mm, whereas α is the slope of this line. The maximal amplitude (MA) is the highest amount of movement that the pin will obtain, which corresponds to the maximum stiffness of the clot. After clotting (presumed to complete at MA), continued motion of the cuvette will cause some lysis of the clot, giving an indicator of how much the clot will dissolve. This time to lysis (TTL) is the time it takes the pin to begin moving 2 mm less than the MA, although this parameter has been shown to represent clot retraction in a platelet count-dependent manner for platelet-rich plasma (PRP) 12. ROTEM uses similar but not identical measurements, so care should be used in comparing these numbers 8. Also, the standard practices, materials and methods used in thromboelastography have changed with time, so caution must be used in comparing results from different time periods 8. Finally, the mechanical response of clots to different agonists and stimulants to blood can be studied with thromboelastography.

#### Bulk platelet contraction

Direct measurements of platelet contractile force are often evaluated from clots *via* a force transducer. In a simple but elegant method described by Cohen *et al*., a clot strip is formed from human PRP and thrombin, which is then attached to a rigid wall on one side and a force transducer on the other 13, 14. The force transducer measures isometric load when the platelets are activated. In another more commonly used method, the downward force is measured as a clot is formed from whole blood between two plates, one of which is connected to a force transducer 15, 16. Both methods are used on macroscopic samples, with the second having the benefit of using whole blood and is therefore more suited for clinical applications. However, these methods have low throughput 15 and there is a possibility that the macroscopic size-coupled effects of passive fibrin elasticity and active platelet forces may convolute contractile force measurements 17.

### Haemostatic diseases & correlations with macroscale mechanics

From a clinical perspective, using the macroscale clot mechanics of a patient's blood can be difficult to interpret as there are many conditions which can cause the same output. However, these tools have provided invaluable insight into how biological and mechanical conditions are dependent on one another.

#### Changes in physiological shear stress affect clot mechanics

Using various types of rheometers, early studies found that shear stress caused by flowing blood affects the stiffness, or elastic modulus, of blood clots. Glover *et al*. found that as PRP was exposed to increasing shear stress, the maximum elastic modulus of the clot formed after 5 min. decreased by a factor of 4 over shear stresses ranging from 30 to 150 dynes/cm^2^ in a series of experiments utilizing a rheogoniometer 18.

#### Abnormal coagulation time is associated with additional complications and is affected by clot composition

The ability to assess coagulation from beginning to end with a simple graphical output allowed thromboelastography to be utilized in clinical settings. Hypercoagulable states occur when time to clotting is faster than normal, and is identified by decreased clotting times, increased α and increased MA values. Hypocoagulation occurs when clots form slower than normal and are identified by increased clotting times, decreased α and decreased MA values 10. Both states are associated with changes in the mechanical properties of clots and overall changes in viscoelastic properties of blood. Many studies correlate hyper- and hypocoagulable states with changes in clot stiffness as measured by TEG. For example, in critically ill patients with sepsis, which often leads to organ failure caused by thrombosis, significantly higher MA values were obtained with TEG when compared with controls 19.

Hypercoagulability and hypocoagulability has been explored in relation to pregnancy. In a study by Sharma *et al*., the coagulability of pre-eclamptic women was investigated. Their results showed that women with mild pre-eclampsia were hypercoagulable as demonstrated by the higher MA values. However, when testing women with severe pre-eclampsia with thrombocytopenia (low platelet counts), the measured MA values were significantly lower than both normal and mildly pre-eclamptic women, showing a transition to a hypocoagulable state 20. Women with a history of multiple miscarriages were also tested for variations in coagulability against non-pregnant women with no history of miscarrying in a study conducted by Rai *et al*. The women who became pregnant were divided into two groups: (i) one that subsequently miscarried again and (ii) one that resulted in live births. Pre-pregnancy MA measurements of women in group 1 were significantly higher than that of women in group 2 and the non-pregnant controls. This result indicated that women experiencing recurrent miscarriage are in a hypercoagulable state outside of pregnancy, and that this state may be a sensitive predictor of future miscarriage 21.

Thromboelastography is also widely used to monitor patients during surgery. In a study by McCrath *et al*., the MA of patients was measured after surgery, and the incidence of myocardial infarction was compared for persons with higher MA values. Of 240 patients tested, 10 had post-operative thrombotic complications. Eighty per cent of patients with post-operative thrombotic complications had abnormally high MA values (>68), and 75% of that group eventually suffered from myocardial infarction. The results of this study indicate that a hypercoagulable state following surgery may be associated with post-operative myocardial infarction 22. The hypercoagulable state has associations with cancer. In a correlative study by Francis *et al*., blood was collected from healthy volunteers, breast and colorectal cancer patients and patients with benign breast or colorectal cancer and analysed with TEG. It was found that nearly half of the patients with colorectal cancer had significantly higher MA values, whereas close to 10% of the patients with breast cancer had significantly higher MA values. However, there were no significant differences in coagulability of patients with benign breast or colorectal cancer 23.

#### Mechanical clot strength is affected by numerous biochemical and mechanical factors in disease states

From numerous TEG studies, it was found that biochemical and even rheological factors affected mechanical clot strength. In patients with peripheral arterial disease (PAD), which is correlated with increased platelet activation, it was found that PAD patients had significantly higher MA values compared with control blood. Additional studies were carried out using tirofiban, a GP IIb-IIIa receptor antagonist, to further test the contribution of platelets to maximum clot stiffness. Findings showed that when the GP IIb-IIIa receptor was blocked, the MA measurements significantly dropped for both PAD patients and normal blood, indicating lower clot stiffness and a hypocoagulable state 24. Class I antagonists of the GP IIb/IIIa receptor (slow platelet dissociation rates owing to increased receptor occupancy) were also found to have a greater inhibitory effect, resulting in a lower elastic modulus, than Class II antagonists (fast platelet dissociation rates owing to relatively lower receptor occupancy). This result shows that binding kinetics may also play a role in the stiffness of clots. In addition, platelets were found to augment clot strength by nearly eightfold under shear when comparing PRP with PPP using TEG 25.

Previous studies on clot formation have shown that the mechanical properties of clots are related to thrombotic or haemostatic diseases 26. For example, clots are 50% stiffer and more resistant to dissolution in young patients with post-myocardial infarction 27 than clots from healthy controls. In addition, mechanical properties of clots formed from peripheral blood are altered in patients who develop idiopathic thromboemboli or acute ischaemic strokes compared with those from healthy controls 28, 29.

#### Mechanical clot strength and structure is affected by clot composition

The structure and composition of blood clots could affect the mechanical properties of blood clots formed *in vitro*. Riha *et al*. performed experiments using a rotational rheometer to observe how the elasticity and fracture strain of clots were affected by varying haematocrit compared with PRP and platelet poor plasma (PPP). PRP clots resulted in the highest elastic modulus followed by the PPP clots. With increasing haematocrit, it was found that the stiffness of the clot was actually lowered while the force required for clot rupture was increased. This result was postulated to be a result of larger pore sizes and decreased numbers of cross-links because of the inclusion of erythrocytes, resulting in a less dense fibrin network 30. By combining microscopy with bulk techniques, Gersh *et al*. found that fibrin clots with varying red blood cell (RBC) concentrations exhibited biphasic behaviour of the fluid and elastic properties. Both the fluid and elastic properties of the clot increased in parallel before peaking at a 10% concentration of RBCs, but higher concentrations of RBCs actually showed a decrease in mechanical properties 31.

Tynngård *et al*. conducted a comprehensive study using a free oscillating rheometer to analyse the effects of different blood components on clot elasticity. In addition to further validating that clots formed from PRP were stiffer than those with high concentrations of RBCs, findings showed that increased fibrinogen and platelet concentration also contributed to higher G' values. Inhibition of the GPIIb/IIIa receptor with abciximab to prevent platelet activation also showed significant decreases in G' to levels close to PPP. However, it was found that PPP-formed clots had the lowest modulus of elasticity in contrast to findings by Gersh *et al*., which stated that PPP-formed clots still had a higher elastic modulus than plasma with different concentrations of RBCs, although this was addressed as an artefact caused by differences in measuring ranges of the equipment used in each study 32.

### Macroscale mechanical clot changes associated with bleeding disorders

#### Type II diabetes mellitus

Type II diabetes mellitus is a metabolic disease that develops from insulin deficiency or resistance. Patients with Type II diabetes mellitus are at an increased risk of cardiovascular and peripheral vascular disease 33. Using techniques such as TEG/ROTEM discussed in the previous sections, researchers used patient samples to observe macroscale differences between healthy and diabetic blood. Patients with diabetes were found to have increased coagulability and stiffer RBCs, indicating that diabetic patients may have differential platelet activity and blood rheology.

In a study conducted by Yurekli *et al*. using ROTEM, it was found that patients with diabetes had increased maximum clot firmness when coagulation was activated with the extrinsic pathway. However, increases in coagulation and clot formation times accompanied by a decrease in α angle were observed when coagulation was initiated through the intrinsic pathway 33. In another ROTEM study by Feuring *et al*., it was found that patients with both coronary artery disease (CAD) and Type II diabetes mellitus had increased maximum clot elasticity values compared with patients with only CAD. These results were obtained for activation using both the intrinsic and extrinsic pathways, indicating that diabetes mellitus may lead to a pro-coagulable state 34. The P2Y_12_ receptor, the platelet receptor for ADP, promotes pro-coagulant activity, and clopidogrel is an antagonist of P2Y_12_. Diabetic patients with low clopidogrel response were also found to have faster R times and time to maximum thrombin generation compared with diabetic patients with optimal response to clopidogrel 35. These results suggest that there may be subpopulations of diabetic patients with varied levels of platelet activity. Finally, using ektacytometry and micropore filtration, a technique that correlates cell deformability with the time taken to cross through a filter, Shin *et al*. found that RBC deformability was significantly decreased in diabetic patients with an inverse correlation with glucose levels 36. Together, these results show that macroscale mechanical properties of blood and clot quality in diabetes mellitus patients are shifted towards a pro-coagulant state, which may explain why patients suffering from this disease are more likely to have cardiovascular complications such as stroke.

#### Haemophilia

Haemophilia is a genetic bleeding disorder that results from either a defect or deficiency in clotting Factor VIII (Type A) or Factor IX (Type B). This results in extended bleeding times and haemorrhaging. Treatment for this disease typically involves infusion of the deficient clotting factor, although some patients eventually develop antibodies against the foreign clotting factors. In those cases, so-called ‘bypass agents’ such as recombinant Factor VII (rFVII), which replenish clotting factors that are downstream of the deficient factors in the coagulation reaction, are typically used. Several studies have been conducted using haemophilic patient samples with macroscale measurement techniques such as TEG/ROTEM to detect differences in clot mechanics, as well as to analyse how normal clot mechanics can be restored using different therapies.

Results from several studies using TEG/ROTEM showed that patients with haemophilia had much longer clotting times, lower maximal amplitude indicating decreased clot stiffness and slower clot initiation times 37–45. In a study by Ghosh *et al*., it was found that TEG could be used to characterize different subgroups of patients with haemophilia between those demonstrating hypercoagulable patterns, hyperfibrinolytic patterns, inability to form clots and variable clot initiation times. A similar study by Viuff *et al*. used tissue plasminogen activator with TEG to measure differences in MA between severe and mild haemophilia patients 37. These changes in mechanical coagulation parameters correlated with clinical manifestations, and could be used to determine the severity of a patient's condition. In addition, TEG profiles of clinically severe groups were able to be improved with the use of epsilon-aminocaproic acid (EACA), which is an antifibrinolytic 38. Treatments with FVIII or rFVIIa were also able to restore TEG traces back to normal in Type A haemophilia patients 39. In a comparison between NN1731 and rFVIIa for bypass therapy, it was found that NN1731 produced more pronounced results. Without treatment of a bypass agent in haemophilia patients with inhibitors, it was found that platelet contractile force (PCF) and clot elastic modulus (CEM), a measurement of clot stiffness obtained by the Hemodyne Hemostasis Analysis System, were decreased while force onset time (FOT) was increased. Treatment with a bypass agent to restore clotting function back to normal increased the PCF and CEM with increasing concentration while the FOT decreased 40. Solulin, a soluble form of thrombomodulin, was also shown to increase clot strength in both haemophilic dogs and humans at low concentrations as determined by ROTEM analysis 42. Personalized treatments were also developed for haemophilia patients on bypass therapy using FEIBA or rFVIIa. Before treatment, clotting times were much longer while the α angle, MA and elasticity were immeasurable. After treatment, clotting times decreased while the α angle, MA and elasticity increased. These changes in mechanical properties were able to be used to determine proper doses of bypass agents and to monitor their efficacy 43. In addition to genetic predisposition, haemophilia can sometimes be acquired as seen in a case study by Spiezia *et al*. Using ROTEM, doctors were able to detect a hypocoagulable state in an elderly woman who experienced life-threatening bleedings during surgery. From the thromboelastogram, it was determined that the woman had developed acquired haemophilia A as seen by the decreased MCF and increased CT. FVIII treatment and continued monitoring of the woman's coagulability were able to successfully increase her MCF and decrease her CT 44.

#### von Willebrand disease

von Willebrand disease (vWD) is another genetic blood disorder that results from qualitative or quantitative defects of vWF. vWD is divided into three different types: Type I is associated with partial quantitative deficiencies and is most common. Type II results from qualitative deficiencies, and is split into Type 2A, 2B, 2M and 2N. Type III is a quantitative deficiency, although vWF is nearly absent in the blood in these patients 46, 47. Ristocetin is an antibiotic that causes vWF to bind to platelets, resulting in platelet agglutination. The agglutinated platelet clumps are then cleared from the circulation, removing them from the clotting reaction 46. Ristocetin is used clinically to assess vWD as platelets will not agglutinate because of the lack of vWF in the blood of vWD patients, whereas agglutination will occur in normal blood.

In a study conducted by Topf *et al*., TEG was used to differentiate between healthy and vWD blood. By taking TEG measurements with blood pre-incubated with ristocetin and then comparing it with TEG traces from blood without ristocetin, the group was able to determine if test participants had vWD. For normal persons, addition of ristocetin lowered clot strength because the agglutination of platelets removed platelets from the coagulation cascade. In patients with vWD, the TEG traces were only slightly inhibited because addition of ristocetin did not affect platelets because of the lack of vWF 46. This technique demonstrates the importance of platelets in maintaining clot mechanical properties and shows how the understanding of clot mechanical properties can be exploited to diagnose blood disorders. Another vWD study using TEG by Guzman-Reyes *et al*. showed that patients with vWD had decreased clotting times, although treatment with desmopressin could normalize this defect 48.

Whether the changes in mechanics are the cause for the observed pathological effects or if the disease states cause changes in clot mechanics remains unknown, but it is evident that proper haemostasis involves finely tuned interactions between biochemistry and biomechanics. Clot elasticity is a major component of haemostasis at the macroscale. If a clot is unable to form or lacks the mechanical fortitude to resist shear stress from circulating blood, then excessive bleeding will occur. If the viscoelastic properties of blood are such that it is in a hypercoagulable state, stiff clots will form and be more likely to lead to thrombosis because of increased difficulty in clot dissolution. As it has been shown that factors such as platelet, RBC and fibrinogen concentrations have some contribution to the mechanical properties of blood clots, it is equally important to understand how mechanics also regulate haemostasis at the single-cell level.

## Microscale biomechanics in haemostasis

Although mechanical stimuli are constantly imparted onto blood cells and plasma proteins in circulation at the bulk ‘macro’ level, many aspects of these haemostatic interactions occur on the microscale. At this scale, shear stress is still a major regulatory factor of mechanotransduction, the process by which external biomechanical stimuli are transduced into biological signals. In the context of blood cells in haemostasis, shear stress-mediated mechanotransduction is critical for inducing biochemical changes, cell–cell interactions and fibrin structure reorganization. Platelets specifically have been found to be remarkably mechanosensitive 49–51, and the role of platelet mechanotransduction in haemostasis will be discussed briefly in this section. In addition, individual cell stiffness contributes to blood rheology and viscosity, which was shown in the previous section to have an effect on the mechanical properties of clots 52. The contraction of platelets is also important in the stabilization of clots for mechanical strength. Characterization of the forces involved in platelet contraction has been investigated by a few groups, and will be discussed in this section. Finally, some of the diseases associated with changes in these single-cell parameters will be reviewed.

The macroscale techniques discussed in the previous section have improved our understanding of haemostatic processes, but the lack of single-cell resolution places a limit on the investigation of microscale interactions that occur in haemostasis. Noting that the clot mechanical structure is often dependent on the ratios of components, it is important to gain an understanding of how each of these components is affected by mechanical stimuli. It is also important to understand how and if the mechanical properties of these components change in response to different chemical and physical conditions. By gaining an understanding of different cell populations, it will be possible to better understand the macroscale effect on clot strength. Current research has shown that each of the components may have a complex and varied reaction to different stimuli. Hence, a more complete understanding of the components of clots will lead to a better understanding of the effects that different stimuli may have on clot mechanics.

To study these interactions at such small scales, researchers have utilized microfluidics, microtechnologies as well as high-resolution techniques such as atomic force microscopy (AFM), micropipette aspiration and optical tweezers to measure the mechanical properties of individual cells and fibrin networks. These techniques will be briefly reviewed in the following sections, as well as their findings and contributions to our working knowledge of haemostasis.

### Tools for microscale measurements

**Fig. 3:**
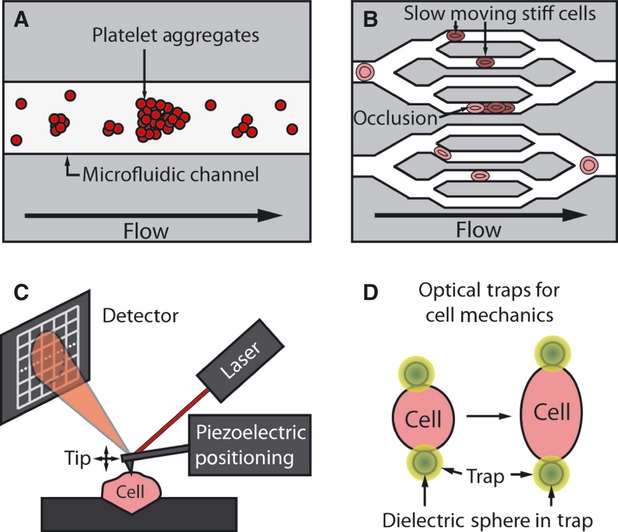
(**A**) Microfluidics can be used to control shear flow of platelets to study platelet aggregation using small sample volumes. (**B**) Microfluidic systems can mimic the hemodynamic and geometric environment of the microvasculature. Stiffer cells move more slowly through the system, and can occlude channels. (**C**) Atomic force microscopy gives detailed measurements of cell stiffness, and can be used to locally probe molecular interactions. For non-adherent cells, some physical or chemical trap is required to hold the cell in place during probing. (**D**) Attaching dielectric spheres to cells and using them in conjunction with an optical trap enable cell mechanical measurements to be made by stretching the cells and measuring the required force needed for a given deformation.

For microscale measurements, higher resolution must be used. Microfluidics allow for controlled laminar flow using small volumes of blood. Clever design considerations can also be used to exploit hydrodynamic forces on the cells to characterize differences in mechanical properties such as cell membrane stiffness. For example, stiffer cells tend to have longer transit times through microchannels. Atomic force microscopy and optical tweezers are similar techniques that allow forces and deformations on the picoNewton and nanometer scale, respectively, to be measured. This attribute makes AFM and optical tweezers useful tools for both micro- and molecular-scale measurements. These techniques are illustrated above in Figure 3.

#### Shear microfluidics

Microfluidics offer a convenient method to analyse blood as it requires relatively small volumes of blood or sample, allow for variable shear rates and the fluid mechanical environment of many different channel designs have already been well characterized 53. The basic unit of a microfluidic system is a channel, which is often created using soft lithography. More in-depth descriptions of the fabrication process in soft lithography are described elsewhere 54–56. Poly-dimethyl-siloxane (PDMS) is the typical elastomer used to create these channels. Fluidic ports have to be pierced into the channel before bonding, and are normally made smaller than the intended tubing for an interference fit. The channel is completed upon covalent bonding to a glass or other PDMS substrate by exposing both surfaces to be bonded to oxygen plasma. Shear stress within the microchannel can then be controlled depending on parameters such as the channel aspect ratio and flow rate.

#### Microfluidic deformation assays

Microfluidic deformation assays work on the same basic principles as shear microfluidics, and are used to characterize the deformability of an individual cell by analysing the cell's ability to squeeze through a channel that is smaller than the cell diameter. Depending on how deformable the cell is, it will either traverse through or obstruct the channel. As PDMS is transparent, the movement of the cell through the channel can be observed, and parameters such as entry time 57, transit velocity 57 and total transit time 58 can be recorded to characterize the deformability of the cell.

#### Microstructures

Another method that utilizes soft lithography relies on the mechanical properties of large numbers of repeating microposts, which essentially act like an array of cantilevers to measure cell traction forces 59. However, the posts are vertically oriented, and an adhesive ligand is usually coated on top of the posts. This technique works on the same principle as conventional traction force microscopy which measures the deflection of beads embedded in a gel to calculate the forces exerted by a cell as it pulls on the substrate 60. When a cell attaches to the end of a post, it will apply a traction force, which will cause the post to deflect. This deflection can then be measured to calculate the traction force. This method is suitable for platelets as they are adherent cells. The deflection of the post is directly related to its spring constant, which can be tuned by modifying the geometry and material selection.

#### Atomic force microscopy (AFM)

Atomic force microscopy is a high-resolution technique which utilizes a scanning cantilever that is moved with nanometer precision through specialized piezoelectric elements. An AFM probes the surface below the cantilever tip for information related to the mechanical properties and surface topography. Force measurements can be made by modifying the system to use a cantilever with a known spring constant, which will deflect linearly with applied force over small amplitudes. The exact applied force is measured by measuring the deflection. Force measurements on the order of piconewtons (pN) are possible with this technique. Mechanical parameters such as the modulus of elasticity and viscoelastic properties can also be extracted from an indentation measurement, although a mechanical model is needed, such as the Hertzian mechanics equation 61, 62.

#### Optical traps

Optical trapping was originally reported by Ashkin 63, 64, who also pioneered the use of the technique with living cells 65. In this technique, radiation pressure is used to trap and move dielectric particles. A laser is commonly used in conjunction with a microscope objective to focus the laser onto the sample plane. Successful optical trapping relies on the use of a beam with an intensity gradient. Light that is passed through the particle is refracted and changes direction, imparting some momentum to the particle. The gradient intensity ensures that the particle ‘feels’ different forces as it traverses through the beam so that it has a tendency to return to the centre of the beam. As an applied force will displace the particle from the centre of the beam, the trap itself may be modelled as a spring. Therefore, by measuring the displacement of the particle from the beam centre, applied force may be calculated, similar to AFM. The precision of the trap depends on the associated spring constant and the resolution at which displacements may be measured. The forces applied by light are quite small, resulting in a much smaller spring constant in optical traps compared with AFM. The ability to make subnanometer displacement measurements makes optical traps a frequently used tool to measure the properties of individual biological molecules 66–69.

Dielectric spheres are typically attached to the object of interest, and manipulated using laser tweezers to measure the mechanical properties of both cells and single molecules 70, 71. More detailed information on the use of optical traps is described elsewhere 72. Mechanical properties such as the shear modulus 73, non-linear elastic properties 70 and viscoelastic properties 70 have been measured using these techniques. Large forces of up to 400 pN have been created to stretch cells to measure non-linear and viscoelastic properties 74. In all cases, a mechanical model of the deformation is required *a priori* to determine these properties.

### Mechanotransduction of platelets in haemostasis

Platelets are constantly exposed to shear stress in the circulation, and elevated shear stress has been shown to initiate a cascade of biological responses involved in haemostasis 75. Several integrins on the platelet surface allow them to actively respond to shear stress and subsequently trigger events such as aggregation, activation and cytoskeletal reorganization. Studies by Feng *et al*. have showed that mechanical forces from shear stress in the circulation directly regulate platelet function through integrin α_IIb_β_3_. Dissociation of cytoskeletal regulatory elements such as α-actinin from the β_3_ tail was observed under elevated shear stress, further supporting the role of shear stress in platelet aggregation and activation 76. GPIb, another mechanosensory receptor on platelets, has been thought to be the primary receptor involved in mechanotransduction of platelets as it responds to increased shear with enhanced adhesion and signalling and forms a complex with von Willebrand factor. However, Goncalves *et al*. found that platelets respond to sudden increases in shear in an α_IIb_β_III_-dependent manner, and suggest that these temporal shear gradients may play a key role for platelet activation. In addition, they also show that P2Y receptors may also be important for integrin mechanotransduction in platelets 50. The proteins involved in integrin mechanotransduction are also important for platelet aggregation and the formation of stable clots. Kasirer-Friede *et al*. found that adhesion- and deregulation-promoting adapter protein (ADAP) promotes F-actin assembly and contributes to the spreading of platelets and formation of stable thrombi under shear. ADAP−/− mice were unable to form stable thrombi, and platelet spreading to fibrinogen was reduced for ADAP−/− *ex vivo*. However, these changes were not observed under static conditions, demonstrating the mechanosensitivity of this adapter protein 49. Observing the single-platelet and bulk clot changes induced by the response of these mechanosensitive receptors is important for better understanding haemostasis and thrombosis, but the entire picture cannot be unveiled without better understanding how mechanics directly impact these receptors at the molecular scale.

### Haemostatic diseases & correlations related to microscale phenomena

Using the aforementioned tools has enabled researchers to study the individual components of clots. By gaining an understanding of the components, better models may be constructed which explain how the macroscale clot mechanics are affected by each constituent.

#### Fibrin is a complex mechanical material with properties which vary by scale, force and shear stress

Fibrinogen is a hexameric plasma protein composed of a pair of three peptide chains designated Aα, Bβ and γ. At each end of the protein are the globular D regions comprised of the β and γ nodules. Triple α-helical coiled coils connect the D regions to the central, globular E region, which contains the two pairs of fibrinopeptides A and B. In haemostasis, activated thrombin removes fibrinopeptides A and B, exposing the A and B knobs in the central E region and converting fibrinogen to fibrin 77.

In addition, blood flow during fibrin formation also impacts the structure of the fibrin network. Fibrin networks formed under static conditions exhibit isotropic fibre distribution, and networks formed under flow are anisotropic, with fibres aligned with flow vectors 78, 79. The alignment of fibrin networks under flow indicates that there is macroscale physics involved related to the shear rates (modelling venous – 10–100/sec.; and arterial – 500–1500/sec. shear rates) on the fibrin network. Varju *et al*. have investigated the impact of longitudinal stretching on fibrin and found that the stretched fibrin structure hinders both stages of fibrinolysis, plasminogen activation and fibrin lysis 80. This indicates that there is an optimal diameter and length of each fibrin, which occurs naturally *in vivo* allowing for fibrinolysis and fibrin lysis to transpire.

The deposition of fibrin has been found to decrease with increasing shear rates when blood is flown over rabbit subendothelium 81. In addition, by quantifying the concentration of fibrin monomers after flowing plasma over an endothelialized extracellular matrix, it was posited that this lack of deposition at high sheer rates was caused by impaired polymerization of fibrinogen 82. These studies corroborate clinical evidence that venous clots (low shear) are mostly fibrin, whereas arterial clots (high shear) are platelet-rich.

Neeves *et al*. utilized a microfluidic device in which wall shear rate and thrombin gradient are independently controlled to elucidate the relation between shear rate and thrombin concentration on fibrin deposition. At a constant thrombin gradient of 10^−12^ nmol/μm^2^ sec., they found that high shear (25/sec.) resulted in a dense fibre mat, whereas low shear (10/sec.) resulted in large, mature fibres. At the highest shear tested (100/sec.), SEM images revealed a thin fibrin film with no discernible fibres. This matched simulations predicting that at high shear rates, the transport of thrombin would be convective in nature, and thus thrombin concentration would be high enough to cause fibrin polymerization only near the source of thrombin. As the concentration of thrombin increases to 10^−11^ nmol/μm^2^ sec., however, the effect of high shear (25/sec.) on fibrinogen polymerization is mitigated, and the fibrin clot occluded the channel. This shows that the polymerization of fibrinogen is highly dependent on both thrombin concentration and shear rate, and that the velocity field causes a reduction in monomer exposure to thrombin, which thereby impairs fibrin deposition 83. The properties of fibrin build upon one another from the molecular to the macroscale. From an applied shear at the macroscale, fibrinogen monomers unfold at the molecular scale, allowing individual fibrin strands at the microscale to align in the direction of the applied strain, which manifests as a stretching of the fibrin network at the macroscale 84.

#### Platelets have contractile properties which are varied and change with the mechanical microenvironment

Atomic force microscopy has been adapted to take measurements of single-platelet contraction forces by Lam *et al*. By placing a thrombin-activated platelet between a fibrinogen-coated AFM tip and a fibrinogen-coated glass surface, single-platelet contraction forces were measured. The deflection of the AFM tip was measured until the force of the cantilever caused contraction to stall. Findings showed that platelets contracted almost instantaneously upon coming into contact with fibrinogen, reached maximum contraction force within 15 min. and had an average maximum contractile force of 29 nN. Furthermore, platelets were found to be capable of exerting forces up to 79 nN, which exceeds those of muscle cells when accounting for the volume difference. Additional experiments were also conducted to simulate platelet contraction on substrates with different stiffness by using AFM cantilevers of different stiffness. It was found that platelets exhibited higher contraction forces as substrate stiffness increased 85.

Platelet contractile force can also be measured microscopically using flexible post sensors 17. In this method, platelets are allowed to adhere to silicone elastomer microposts. As a clot begins to form and the platelets contract, the posts are pulled towards each other. Using fluorescence microscopy, the deflection of the microposts caused by the contraction of a microthrombus was measured to determine contractile force with various concentrations of thrombin. Increasing thrombin from 1 to 10 U had the effect of increasing the average contractile force. The average force per platelet was found to be 2.1 + 0.1 nN after 1 hr 17.

#### The location of platelet thrombus formation is affected by local shear gradients

Another study by Nesbitt *et al*. utilized specially designed microfluidic channels that mimicked stenoses to observe the effects of shear microgradients on platelet aggregation. The results of this study showed that forming thrombi created low-shear zones at the downstream face, and discoid platelets preferentially adhered to the low-shear zones. Furthermore, the stability of platelet aggregates was found to be dependent on the dynamic restructuring of membrane tethers 86.

### Microscale changes are also observed for various bleeding disorders

#### Type II diabetes mellitus

Using AFM, studies have shown that some of the complications associated with diabetes may be caused by increased RBC membrane stiffness. Chen *et al*. found that the average stiffness of diabetic RBCs was significantly higher than those of healthy non-obese RBCs 87. Jin *et al*. examined the nanoscale adhesive forces and stiffness of RBCs in diabetic patients of varying ages. Results showed that both the nanoscale adhesive forces and stiffness of RBCs was significantly higher in elderly diabetic patients compared with healthy controls of young and elderly age groups 88. Another study by Starodubtseva *et al*. found that diabetic patients have a heterogeneous population of RBCs compared with healthy controls. Diabetic patients were found to have poikilocytosis and anisocytosis, RBC cytoskeletal reorganization and modified membrane mechanical properties 89. These microscale changes may potentially affect the overall blood viscosity, which can lead to changes in shear stress and ultimately clot formation.

#### Haemophilia

Using microchannels, Ogawa *et al*. investigated the effect of shear rate on thrombus formation in haemophilic patients. FVIII−/− mouse blood and human blood with anti-FIXa to simulate haemophilia B was perfused over collagen-coated microchannels at venous and arterial shear rates. The time taken to observe pressure changes of 5 and 40 kPa caused by thrombus formation was recorded. The time required to observe a pressure change of 40 kPa essentially represents the time to advanced thrombus formation. In the FVIII−/− mouse blood, the advanced thrombus formation time was significantly increased compared with normal controls at venous shear rates. Addition of human FVIII was able to normalize the advanced thrombus formation time. In the simulated human haemophilia B blood, the advanced thrombus formation time was again significantly increased when compared with normal healthy controls at venous shear rates. However, both samples did not show significant differences when flowed at arterial shear rates 90. This study shows that platelet aggregation, a component necessary for the formation of a stable clot, is inhibited in haemophilia.

#### von Willebrand disease

In a study by Hansen *et al*., microfluidic channels were used to assess platelet function in patients with vWD. After flowing blood of patients with vWD over collagen thin films in a microfluidic channel, the surface coverage with platelets of the collagen thin films was measured. Compared with normal healthy controls, the surface coverage under flow was decreased in platelets with vWD, indicating that platelet adhesion and aggregation is inhibited in patients with vWD 91. This result may potentially explain the observations observed at the macroscale described in earlier sections. However, more work at the microscale should be conducted in the individual components of the coagulation cascade such as fibrin formation in vWD patients.

### Biochemical and biomechanical interactions

Biochemical and biomechanical interactions can be studied at the macro- and microscale, but the mechanisms of how these changes result in mechanical alterations remain still unclear. For example, it is known that platelets contract differentially depending on the amount of thrombin used to activate them, or that platelets cannot form stable clots when the GP IIb/IIIa receptor is inhibited with a drug. However, only correlations can be drawn from these types of studies. The true mechanism for what is actually going on cannot be fully understood without exploring haemostasis on the molecular scale.

## Molecular-scale biomechanics in haemostasis

At the smallest scale, the link between mechanics and biochemistry becomes even more pronounced. Structural changes that occur at larger scales necessary for haemostasis are a direct result of signalling cascades that occur at the molecular scale. It is the combined effect of the glycoprotein receptors on the surface of platelets and their interactions with soluble shear-sensitive proteins released by activated platelets and flowing in blood that allow for platelets to generate the adhesive forces necessary to attach to damaged vessel walls. Once again, shear stress is an important regulatory force that in return allows these haemostatic components to induce a biomechanical or biochemical response that propagates to ultimately affect the mechanics at larger scales. In this section, we will discuss how the mechanosensitive molecules of haemostasis such as vWF, fibrinogen and platelet surface glycoprotein receptors are mechanically regulated and their contribution to forming stable cell adhesions for haemostasis. In addition, we will also discuss the mechanical properties of individual fibrin strands, and how they contribute to the overall clot structure. As we are currently at a point of technological limitation to directly measure forces and interaction at the molecular scale, most of the current work at this scale relies on adaptations of the microscale tools discussed in the previous section. The mechanosensitive plasma proteins, vWF and fibrinogen, and their role in haemostasis are discussed briefly in the following sections, as well as some of the clinical findings at the molecular scale of some of the diseases discussed in the previous sections.

### Molecular components involved in haemostasis

#### von Willebrand factor (vWF)

Shear stress plays two critical roles in vWF regulation: (i) Shear-dependent vWF multimer unfolding and following breakdown by a disintegrin-like and metalloprotease with thrombospondin type 1 motif no. 13 (ADAMTS-13) and (ii) High-shear-sensitive vWF binding to the platelet glycoprotein GPIb-IX-V complex.

Monomers of vWF are roughly 280 kD and are linked by disulphide bonds into the multimer which can be up to 20,000 kD 92. These extremely large multimers are called ‘unusually large’ ULvWF, which are produced in endothelial cells and megakaryocytes. They are stored in platelet alpha-granules and Weibel-Palade bodies in endothelial cells. ULvWF is highly adhesive, and once secreted binds to exposed subendothelial collagen at the site of vascular injury, promoting platelet adhesion. As ULvWF has high adhesive potency, in healthy blood vessels, they break down into smaller multimers that are less adhesive, and are therefore less thrombotic, in a shear-dependent manner.

Flow experiments in capillary tubes, parallel flow chambers and AFM show that shear force modulates the conformation of vWF multimers from a globular state to an extended chain, and enhances susceptibility to proteolysis 93–96. A study using microfluidic flow chambers showed that unusually high shear flows (>1,000/sec.) result in an abrupt elongation of vWF fibres in solution possibly because of their large repeating unit size 97. Zhang *et al*. used the optical trap technique to show that tensile forces of 7–14 pN can unfold a single A2 domain of vWF which is cleaved by ADMTS-13 upon its exposure 98. Other recent studies using optical traps/tweezers have provided more insight into the mechanical properties and stability of vWF at the molecular level 99, 100.

#### Fibrinogen/Fibrin

Of interest when considering the molecular aspects of haemostasis are the mechanical properties of individual fibrin monomers that give rise to clot elasticity and permeability. Using atomic force microscopy, it has been found that covalently cross-linked fibrin monomers can be stretched to 2.8 times their original length and still recover elastically, and 4.3 times their original length before rupture. These fibres are thus highly elastic as well as highly extensible 101. The molecular underpinnings of this elastic nature have been probed by forcefully unfolding fibrin using single molecule AFM. Brown *et al*. found that oligomer unfolding gives rise to a periodic sawtooth pattern with an average peak force of 94 pN that can be attributed to the unfolding of the triple helix coiled coils. This unfolding would account for up to twofold strain in fibre extensibility 102. In a later study, Brown *et al*. posited that the linear response of fibrin fibres at a strain of ∼0.15 is caused by the initial straightening of protofibrin to align with the strain, followed by unfolding of the coiled coils up to a strain of 1.2. This linear relation continues until strain hardening occurs and the strain increases more rapidly with the unfolding of additional fibrin molecules 84. Zhumorov *et al*. continues the previous findings using AFM coupled with graphics processing units (GPU)-based computational acceleration force unfolding simulations to investigate the deterministic hierarchy of the consecutive unravelling of structures within the protein. They conclude that extensibility of fibrin starts with the α-helical coiled coils which serve as molecular capacitors that extend and contract reversibly, followed by force-induced release of the β-strands, and the eventual dissociation of the γ-nodules. These results hold from single fibrinogen molecules to polymeric fibrin, suggesting that unfolding happens in the same structural components at each scale 103.

### Molecular-scale changes in mechanics associated with bleeding disorders

Several molecular-scale diseases exist, which have provided researchers with model systems to better understand the molecular interactions that contribute to haemostasis and thrombosis. These are usually genetic defects that result in the loss or malfunction of a molecular component necessary for haemostasis. Glanzmann thrombasthenia is an autosomal recessive disease that is a result of abnormalities of the αII_b_β_3_ integrin, which is a mediator of platelet aggregation. This impaired binding of adhesive proteins affects proper thrombus formation during vascular injury, and results in excessive bleeding 104. One study by Filomena *et al*. showed that patients with Glanzmann thrombasthenia had decreases in the frequency of binding/unbinding events with fibrinogen and force rupture values, which may explain some of the clinical observations 105. Grey platelet syndrome is another genetic bleeding disorder that results in a lack of α-granules, which contain many biochemical factors important to clotting such as platelet factor 4, β-thromboglobulin and fibrinogen. This often manifests as a mild-to-moderate bleeding tendency, although severe haemorrhaging can occur in rare cases 106. Studying these diseases has allowed researchers to better understand how biochemistry and biomechanics contribute to clot dynamics at each scale, although exact mechanisms are still not fully understood. However, these biochemical and biomechanical interactions of platelets are out of the scope of this review. A more comprehensive review on platelet dysfunction by Ramasay *et al*. is suggested for more information 107.

#### von Willebrand disease

In a study by Yago *et al*., it was found that Type 2B vWD patients had prolonged bond lifetimes between GPIbα and the vWF A1 domain. This allows ADAMTS-13 to deplete large vWF multimers and cause the excessive bleeding observed clinically 108. A similar study by Auton *et al*. used AFM to determine how the conformational stability of the A1 domain of vWF was affected in Type 2B and 2M vWD. Findings showed that the molecular-scale changes in these diseases affected the force-dependent dissociation kinetics of the vWF–GPIbα interactions. In Type 2B vWD, the stability of the A1 domain of vWF is decreased, whereas stability is increased in Type 2M vWD. The bond lifetimes were also increased in Type 2B vWD compared with WT A1, whereas Type 2M vWD exhibited shorter bond lifetimes. From these results, it was suggested that stability of the A1 domain ultimately affects the shear required for optimal binding of vWF with platelets. These results also explain the reversed trends of platelet binding seen in the two diseases. Decreased stability of the A1 structure leads to increased GPIbα binding, which leads to spontaneous platelet binding. Increased stability of the A1 domain leads to decreased GPIbα binding, which reduces platelet binding with vWF. Therefore, it was shown that the physical structure of vWF caused by mutations affects the binding stability, which has an effect on binding kinetics caused by rheological shear 109.

## Erythrocytes in haemostasis

Erythrocytes vastly outnumber any of the other blood cells in the vasculature and contribute to the rheological properties of the blood. Recent computational models suggest that stiff cells marginate to the outside of vessel walls caused by hydrodynamic forces, which implicates that stiffer cells may interact with clots more 110–112. Analysis of thrombi composition in acute myocardial infarction also showed that clots can have as high as 20% RBC composition 113. Therefore, this section will focus mostly on factors that cause RBC stiffness to change. Many of the same techniques described above are able to measure RBC deformability, but ektacytometry has also proven to be a useful tool in measuring the bulk deformability of RBCs.

### Erythrocyte stiffness is varied and affected by a number of different diseases

#### Sickle cell disease (SCD)

Sickle cell disease is the result of a recessive genetic mutation that affects the haemoglobin in RBCs. The sickle haemoglobin S (HbS) polymerizes under deoxygenated conditions, resulting in the characteristic sickle shape of the RBCs. These cells tend to be less deformable than normal RBCs, which change the rheological and viscous properties of blood. As a result, several pathophysiological complications arise such as vaso-occlusion or stroke. As sickle cell disease has a genetic basis, two different classes exist: homozygous (HbSS) and heterozygous (sickle cell trait/HbAS) 114.

#### Malaria

Malaria is a disease that is caused by a parasite of the genus *Plasmodium*. Among humans, *Plasmodium falciparum* is the most common infection. Once in the bloodstream, the parasites invade RBCs and divide, ultimately leading to RBC lysis. During the asexual development of *P. falciparum* in the RBC, changes in the spectrin network occur, and can be characterized into three stages: (i) the ring stage that occurs for the first 24 hrs of RBC infection, (ii) the trophozoite stage that occurs 24–36 hrs after invasion and (iii) the schizont stage where the parasite divides, typically for 36–48 hrs after invasion. Several changes in the RBC cytoskeleton and membrane occur, at times even changing from a biconcave shape to a spherical shape 115, 116.

Using a microfluidic device with varying channel sizes to recapitulate different microvessel geometries, Shelby *et al*. characterized the deformability of RBCs in different stages of *Plasmodium falciparum* infection by analysing RBC deformability through channels of different sizes. Findings showed that normal uninfected RBCs were able to deform through channels as small as 2 μm whereas the deformability of infected cells was altered depending on the stage of infection, limiting the RBC's ability to traverse through channels of decreasing diameter. Ring-stage infected RBCs (an early stage of infection) were able to pass through channel sizes of 2–8 μm like normal RBCs, but schizont-stage RBCs (a late stage of infection) could not pass through any channel sizes smaller than 8 μm 117. Exploiting these differences in deformability, Hou *et al*. were able to fabricate a device that could sort infected from uninfected RBCs using the rheological properties of blood that resulted in margination of the infected RBCs towards the outer walls 118.

Other uses for AFM in the study of microscale mechanics of haemostasis include the measurement of elastic moduli of single blood cells. The elastic modulus of sickle RBCs and normal RBCs was measured by Maciaszek *et al*. Results showed that sickle RBCs had a higher modulus of elasticity compared with normal RBCs. Furthermore, deoxygenated SCD RBCs had a much higher elastic modulus compared with oxygenated SCD RBCs 119.

Optical tweezers were used to generate force–displacement curves from RBCs. In different intracellular developmental stages of *P. falciparum*, the shear modulus of RBCs was found to increase up to 10-fold 120. The deformability of RBCs was also measured in SCD patients by Brandão *et al*. to investigate the effects of hydroxyurea treatment. In HbSS patients and HbAS patients, the RBC deformability was significantly lower than the normal controls. With long-term hydroxyurea treatment (6 months+), HbSS patients had RBCs that were similar to normal controls in deformability. This technique was also sensitive enough to detect differences in deformability between HbAS patients and normal controls 121.

Using these controlled fluid microenvironments, studies have shown how shear stress can elicit different responses from blood cells at the microscale. Wan *et al*. showed that RBCs are able to release ATP with a decreasing time delay under increased shear conditions through retraction of the spectrin-actin cytoskeletal network 122. Although this function of RBCs is normally thought to contribute to blood pressure regulation owing to the vasodilatory signalling functions of ATP, other studies have found that ATP can induce platelet aggregation in whole blood 123.

## Concluding statements

As demonstrated in this review, mechanics play an important role in haemostasis and thrombosis. Blood cells and soluble plasma proteins have developed in such a way to withstand shear stress and hydrodynamic forces, but also use these forces to regulate their function and regulate the mechanics of clotting from the molecular to the macroscale. Recall that changes in the mechanical properties of clots at the macroscale have been observed for different diseases and genetic mutations. For instance, clots have been found to be stiffer in diabetes mellitus and softer in haemophilia and von Willebrand disease. Nevertheless, when examining the individual components of clots, which are primarily responsible for their structure and stiffness on the microscale, interesting properties have been discovered. For example, platelets have recently been shown to modulate the applied mechanical force depending on the local microenvironment stiffness. However, the local mechanical environment is partially dictated by the fibrin structure of the clot, which is known to change with shear stress and flow direction. The challenge remains in understanding both the individual responses of cells to mechanical stimuli, and in understanding the combined complex response of the system to mechanical stimuli. Preliminary studies are beginning to show that even spatial aspects of the microenvironment may contribute to the regulation of platelet aggregation 124–126. Ultimately, discoveries on the micro- and molecular scale need to be linked back to the initial clinical discoveries made on the macroscale. There is a great need to understand what the conditions are in different pathologies, which create different mechanical environments. Further research would enable the creation of systematic models which are able to fully predict how clot stiffness will change for various pathological conditions. This is important because we could potentially predict when patients are at risk for stroke and haemorrhaging much earlier. A brief overview of bleeding diseases and their mechanically related clinical manifestations is provided above in Table 2.

**Table 2:** General overview of diseases that affect the mechanical properties of blood, leading to alterations in clot composition or thrombosis

Disease	Cause	Clinical manifestation related to mechanics at macro-, micro- and molecular scales	References
Type II Diabetes Mellitus	Insulin deficiency or resistance	Increased clot firmness and coagulability	33–36
		Decreased RBC deformability	
		Enhanced platelet pro-coagulant activity in patients with suboptimal clopidogrel response	
		RBC membrane stiffness increased	87–89
		Higher nanoscale adhesive stiffness of RBCs in elderly diabetic patients	
		Poikilocytosis and anisocytosis, RBC cytoskeletal reorganization	
		Heterogeneous population of RBCs with varying mechanical properties	
Haemophilia A/B	Defect or deficiency in Factor VIII (A) or Factor IX (B)	Extended bleeding times	37–45
		Longer clotting times	
		Less stiff clots	
		Bulk platelet contractile force decreased	
		Increased platelet aggregation time	90
von Willebrand Disease (Type 1, 2A, 2B, 2M, 2N and 3)	Either qualitative (Type 2) or quantitative (Type 1 and 3) defects of vWF.	Increased bleeding times	46, 48
		Inability to clear out agglutinated platelets with ristocetin. Normal blood experiences a reduction in clot strength when incubated with ristocetin contrary to vWD patients.	
		Surface coverage of platelets on collagen thin films under flow is decreased.	91
		Prolonged bond lifetimes between GPIbα and the vWF A1 domain	108, 109
		The physical structure of vWF owing to mutations affects the bonding stability, which has an effect on binding kinetics caused by rheological shear.	
Hypercoagulable State	Pre-eclampsia, correlation with miscarriage, thrombotic complications after surgery	Clotting occurs quicker than normal, resulting in stiffer clots	19–23
Hypocoagulable State	Critically ill patients with sepsis, severe pre-eclamptic women with thrombocytopenia, Peripheral arterial disease	Increased clotting times	20, 24, 44
		Softer clots	
Malaria	*Plasmodium Falciparum* parasite	Nervous, respiratory, renal and/or haematopoietic complications	115, 116
		RBCs stiffen as the parasite grows	117, 118, 120
		Deformability is decreased	
		Increased shear modulus	
Sickle Cell Disease	Genetic mutation in the haemoglobin gene	Vaso-occlusion or stroke because of increased clotting	114, 119
		Individual cells are stiffer	121
		Deformability is decreased	

Mechanical stimuli induce physical and biochemical changes at all scales, which in turn modulate the mechanics at increasingly larger scales. These factors are intimately tied together, and a balance must be maintained, otherwise pathophysiological effects can result. Haemostasis is a complex process that despite being studied for several decades is still not fully understood. Although several components of haemostasis have been studied individually, there is a missing link which exists because these processes occur over multiple scales. Future studies should focus on not only the smaller scales but also how they tie back into the macroscale changes observed clinically. Although more efforts are being put into understanding the biomechanics of haemostasis, it is critical to understand how these blood cells interact with the mechanical and spatial microenvironment as well. Further investigation of these factors will better elucidate platelet function and the coagulation cascade to characterize the biomechanical factors involved in haemostasis and diseases of clotting and bleeding.
